# Tissue-specific transcriptomes of *Anisakis simplex* (*sensu stricto*) and *Anisakis pegreffii* reveal potential molecular mechanisms involved in pathogenicity

**DOI:** 10.1186/s13071-017-2585-7

**Published:** 2018-01-10

**Authors:** Serena Cavallero, Fabrizio Lombardo, Xiaopei Su, Marco Salvemini, Cinzia Cantacessi, Stefano D’Amelio

**Affiliations:** 1 Department of Public Health and Infectious Diseases, Sapienza University of Rome, Rome, Italy; 2 Department of Veterinary Medicine, University of Cambridge, Cambridge, UK; 3 Department of Biology, University of Naples Federico II, Naples, Italy

**Keywords:** *Anisakis simplex* (*sensu stricto*), *Anisakis pegreffii*, Pharyngeal transcriptome, Pathogenesis, Differential gene expression, Peptidases

## Abstract

**Background:**

Larval stages of the sibling species of parasitic nematodes *Anisakis simplex* (*sensu stricto*) (*s.s.*) (AS) and *Anisakis pegreffii* (AP) are responsible for a fish-borne zoonosis, known as anisakiasis, that humans aquire via the ingestion of raw or undercooked infected fish or fish-based products. These two species differ in geographical distribution, genetic background and peculiar traits involved in pathogenicity. However, thus far little is known of key molecules potentially involved in host-parasite interactions. Here, high-throughput RNA-Seq and bioinformatics analyses of sequence data were applied to the characterization of the whole sets of transcripts expressed by infective larvae of AS and AP, as well as of their pharyngeal tissues, in a bid to identify transcripts potentially involved in tissue invasion and host-pathogen interplay.

**Results:**

Approximately 34,000,000 single-end reads were generated from cDNA libraries for each species. Transcripts identified in AS and AP encoded 19,403 and 10,424 putative peptides, respectively, and were classified based on homology searches, protein motifs, gene ontology and biological pathway mapping. Differential gene expression analysis yielded 226 and 339 transcripts upregulated in the pharyngeal regions of AS and AP, respectively, compared with their corresponding whole-larvae datasets. These included proteolytic enzymes, molecules encoding anesthetics, inhibitors of primary hemostasis and virulence factors, anticoagulants and immunomodulatory peptides.

**Conclusions:**

This work provides the scientific community with a list of key transcripts expressed by AS and AP pharyngeal tissues and corresponding annotation information which represents a ready-to-use resource for future functional studies of biological pathways specifically involved in host-parasite interplay.

**Electronic supplementary material:**

The online version of this article (10.1186/s13071-017-2585-7) contains supplementary material, which is available to authorized users.

## Background

Food-borne diseases are caused by a variety of chemical insults and pathogenic organisms, including parasites, with *Anisakis* spp. being the only fish-borne parasites able to trigger an allergic response in humans [1]. Species of *Anisakis* are indeed responsible for a relatively poorly known food-borne zoonosis, known as ‘anisakiasis’, that occurs in large areas of the globe, including Japan and other easternmost regions, as well as the Netherlands, Germany, France, Spain, Croatia [2] and Italy, amongst others [3].

*Anisakis* spp. (Ascaridoidea: Anisakidae) are nematodes with a cosmopolitan distribution whose life-cycle depends on aquatic hosts [4]. Definitive and intermediate hosts are marine mammals and crustaceans, respectively, while fish and squids can act as paratenic hosts, harbouring infective third-stage larvae mostly in their body cavities. However, larvae are often found in fish muscles (fillets), where they migrate before the death of the host [5]. The occurrence of larval nematodes in fish fillets is of particular medical and economic concern; indeed, beside the effects on the marketability of marine products, *Anisakis* third-stage larvae (L3 s) are the causative agents of a human disease known as anisakiasis. This occurs as a consequence of accidental ingestion of L3 s, and consists of a mild to severe disease classified as gastric anisakiasis (GA), intestinal (IA) and extraintestinal anisakiasis depending on the localization of the larva [6, 7]. In addition, infection with *Anisakis* spp. may cause sensitisation to parasite allergens [8, 9] that, following subsequent exposure, can result in a variety of systemic reactions [10]. Moreover, *Anisakis* spp. have been recently observed in the same localization with gastro-intestinal tumors [11–13]. While, thus far most reports of anisakiasis originate from regions of the world where consumption of raw or undercooked fish is common (e.g. Japan) [3], the global prevalence of gastrointestinal and allergic anisakiasis is likely to be severely underestimated, particularly because of the intrinsic limitations of currently available diagnostic tools.

*Anisakis simplex* (Rudolphi, 1809) (*sensu stricto*) (*s.s.*) is responsible for almost all of the reported human cases of anisakiasis in Japan, whereas its sibling species, *Anisakis pegreffii* Campana-Rouget & Biocca, 1955, is responsible for most cases of anisakiasis in southern Europe [1]. *Anisakis simplex* (*s.s.*) displays higher tolerance for artificial gastric juice and acid [14, 15] and penetrates both agar and fish muscle [15, 16], as well as digestive tract tissues of Wistar rats [17] more efficiently than *A. pegreffii*. Interestingly, recent comparative analyses of the transcriptomes and proteomes of *A. simplex* (*s.s.*) and *A. pegreffii* larvae revealed both qualitative and quantitative differences of the potential allergens responsible for the onset of allergic anisakiasis [18, 19], thus calling on more investigations into potential adverse effects elicited by these two species.

Indeed, in spite of growing concerns for public health due to anisakiasis, the molecular mechanisms responsible for the pathogenicity of *Anisakis* spp. remain largely unknown. Pharyngeal excretory glands of the *Anisakis* larval stages have long been hypothesized to be implicated in such mechanisms through the release of proteolytic enzymes [20–22], and data from other parasitic nematodes of the same superfamily (Ascaridoidea) suggest that peptidases could play key roles in biological pathways linked to fundamental host-pathogen interactions [23]. However, thus far and to the best of our knowledge, no data are available on the molecules transcribed by the pharynx of *Anisakis* spp. The identification of these molecules and the characterization of their expression profiles compared to other larval tissues may provide clues as to their role(s) in biological pathways associated with the pathogenicity of these parasites. Therefore, in the present study, an in-depth analysis of differential gene expression between the whole larva and the pharyngeal tissues of both *A. simplex* (*s.s.*) and *A. pegreffii* was carried out, transcriptomes were obtained and molecules putatively responsible for the pathogenicity of these two species were identified.

## Methods

### Parasite material

**Fig. 1:**
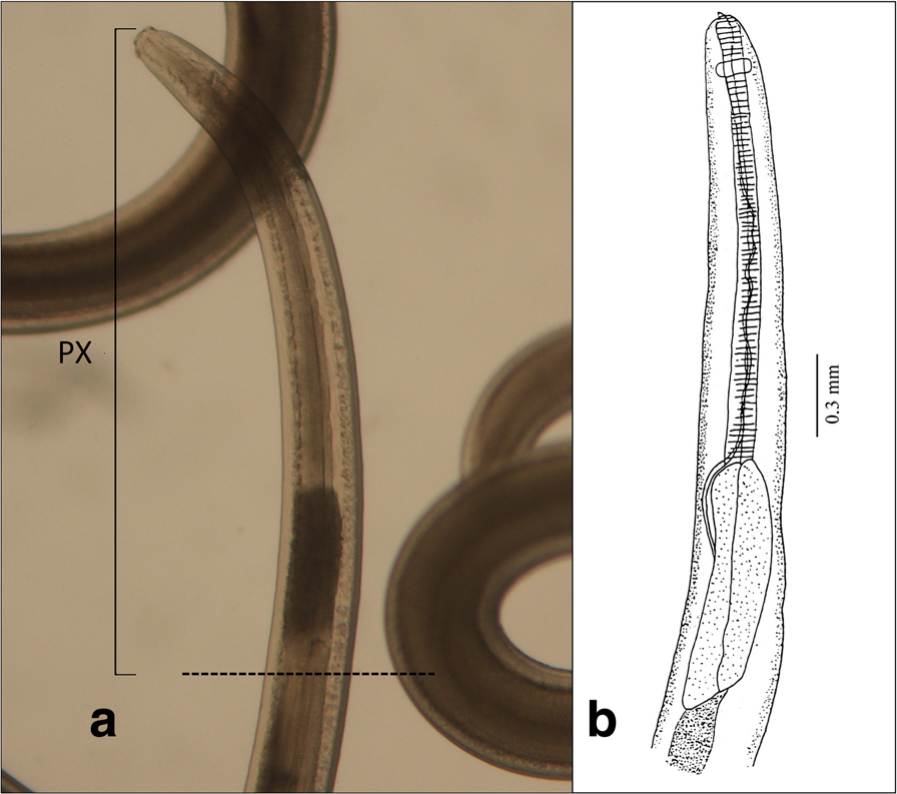
Apical region of *Anisakis* type I larva *sensu* Berland [63]. **a*** Anisakis* spp. larvae. **b** Schematic drawing of the pharynx region, with diagnostic features relative to shape and size. Dashed line indicates the dissection point; bracket with PX indicates pharyngeal region dissected

Three fish species were collected and analyzed between 2015 and 2016: the Atlantic mackerel *Scomber japonicus* from the major FAO 27 fishing area (North East Atlantic region), the European anchovy *Engraulis encrasicolus* and the European pilchard *Sardina pilchardus* from the FAO 37 (Mediterranean). Viscera and fillets of each fish were visually inspected under a stereomicroscope for the detection of *Anisakis* larvae. Parasites were collected, washed and stored in sterile PBS for subsequent dissection of the pharyngeal tissues (defined as the apical portion starting with the linear pharynx and continuing with the globular ventricular oesophageal appendix (see Fig. 1) henceforth designated as ‘PX’). Tissues were dissected under a stereomicroscope and the remainder of the larva (‘WL’) was stored separately for further DNA and RNA extractions (see below). Samples corresponding to PX and WL of each larva were homogenized and processed using the TRIsure reagent (Bioline, London, UK), according to the manufacturer’s instructions, for simultaneous isolation of DNA and RNA, thus overcoming potential biases due to partial removal of larval tissues or organs.

Genomic DNA was used as a template for molecular identification of parasite species, based on PCR-RFLP analysis of the nuclear ribosomal marker internal transcribed spacer (ITS) according to published diagnostic keys [24].

### RNA extraction and quality check

Total RNA was extracted using the TRIsure™ reagent (Bioline) and contaminating genomic DNA was removed using the DNAse in the Turbo DNA-free kit (Life Technologies, Waltham, USA). Three independent biological replicates were analysed, each one consisting of total RNA extracted from three WL and ten PX of each *A. simplex* (*s.s.*) (‘AS’) and *A. pegreffii* (‘AP’), respectively. The quality of the extracted RNA was verified visually on agarose gel, while resulting quantities were measured using the Take3 Module of Synergy HT Multi-Detection Microplate Reader (Biotek, Winooski, USA) and a Bioanalyzer 2100 (Agilent Technologies, Waldbronn, Germany).

### cDNA library construction and RNA-sequencing

RNA strand-specific libraries were created using the New England Biolabs Kit (NEBNext® Ultra™, Ipswich, USA), according to manufacturer’s instructions (Illumina, San Diego, California). In brief, polyadenylated (PolyA+) RNA was purified using the NEB (NEBNext® Poly(A) mRNA Magnetic Isolation Module) from 3 μg of total RNA from each WL and PX sample, fragmented and reverse transcribed into cDNA. Following fragmentation of the mRNA, first and dUTP-based second strand synthesis were carried out, followed by end-repair, A-tailing, ligation of the indexed Illumina adapters and digestion of the dUTP-strand. Ligated products of 150–400 bp were excised from agarose gels and PCR amplified. Products were cleaned using a MinElute PCR purification kit (Qiagen, Hilden, Germany) and the quality of each cDNA library was verified on an Agilent 2100 Bioanalyzer. For RNA-Seq, pooled libraries were single-end sequenced on a HiSeq2500 platform (Illumina) using HiSeq Flow Cell v4 and 125 bp read length.

### Bioinformatics analyses of sequence data

Raw reads were trimmed and filtered for Illumina adaptor sequences, sequences with suboptimal read quality (i.e. PHRED score ≥ 25.0) and sequences shorter than 125 bp using Trimmomatic v.0.36 [25].

For AS, the high-quality single-end reads representing both WL and PX were mapped to the available *A. simplex* reference genome (Assembly GCA_000951095.1) with BWA-MEM version 0.7.12-r1039 [26] with default parameters. Resulting alignments were extracted and information linked to genome regions to which reads were mapped (e.g. intron-exon boundaries) were obtained from the GTF annotation file available from the WormBase repository database at http://parasite.wormbase.org/Anisakis_simplex_prjeb496/. Then, the number of raw reads mapping to each *A. simplex* gene was calculated using the *Rsubread* package in R (https://www.r-project.org/). Transcripts differentially expressed in WL and PX were identified by *DESeq2* package in R (|logFC| > 2). In order to control for errors associated with multiple pairwise comparisons, a false-discovery rate correction (FDR < 0.05) was applied to the data set [27].

For AP, clean reads obtained from high-throughput sequencing of WL and PX were pooled and assembled *de novo* using the Trinity software [28]. Subsequently, reads from each WL and PX were individually aligned to the assembled transcriptome using Bowtie, and the relative expression of each transcript was estimated via the RSEM package. The trimmed mean of M-values normalization method (TMM) was used for cross sample normalization, and the normalized expression of each transcript was presented as transcripts per million (TPM). Assembled transcripts with < 10 aligned reads were eliminated from subsequent analyses (http://deweylab.biostat.wisc.edu/rsem/). Differentially expressed transcripts between WL and PX were identified by *DESeq2* package in R (|logFC| > 2; FDR < 0.05).

Putative ortholog transcripts shared between AS and AP were identified using a reciprocal best hit analysis on the longest ORF, and by retaining only the sequences aligned with a continuous region covering at least 30% of the query sequence [29]. To better define the secretory nature of predicted proteins in these tissue-specific repertoires, we refined the search of predicted signal peptides in both oesophageal and whole body-enriched catalogues. Among enriched subsets, only contigs encoding for peptides starting with Met were retrieved and the probability to carry signal peptides was evaluated using the on-line prediction server SignalP [30].

Transcripts encoding for proteins belonging to five families, i.e. astacins, ShK toxin, EB cystein-rich modules, Kunitz and CRISPs (CAP superfamily), were selected amongst molecules upregulated in the PX of each AS and AP and subjected to further analyses using the BLAST search tool, Clustal Omega, Artemis and domain organization platform available from the EMBL-EBI Pfam website. These molecules were selected based on their documented roles in key mechanisms of host-parasite interactions [23].

For AP, transcript annotation was performed by comparing sequences to data available in the nucleotide sequence collection (Nt) of NCBI and in the non-redundant (Nr) (www.ncbi.nlm.nih.gov) and SwissProt (http://expasy.org/) databases using BLASTn and BLASTx algorithms, respectively. The software Trinotate (Transcriptome Functional Annotation and Analysis) (https://trinotate.github.io/) was used to extract annotation information from the eggNOG, KEGG and Gene Ontology databases using default settings. The coding regions of transcripts were predicted using TransDecoder [28].

## Results

### Samples and species identification

A total of 85 anisakid larvae were collected, i.e. 31 from Atlantic mackerels, 32 from European anchovies and 22 from European pilchards. Of these, 31 larvae collected from the Atlantic mackerel were identified as AS, and 34 larvae collected from European anchovies and European pilchards were identified as AP following restriction analysis with *Hinf*I [24]. Twenty larvae collected from European pilchards were identified as *Hysterothylacium aduncum* and were thus excluded from the study. Three WL and PX samples from each AS and AP were subjected to high-throughput RNA-Seq.

### The *Anisakis simplex* (*s*.*s*.) and *A*. *pegreffii* transcriptomes

A total of 32,859,191 and 37,606,372,125 bp reads were generated for each WL and PX of AS, while 23,397,005 and 36,382,470 reads were generated from WL and PX of AP, respectively (Table 1). Following pre-processing for quality, a total number of 29,011,662 (WL-AS) and 32,853,551 (PX-AS), 21,128,142 (WL-AP) and 32,711,755 (PX-AP) reads were retained for further analyses. Raw reads generated in the present study have been submitted in the Sequence Read Archive (SRA) database of NCBI (http://www. ncbi.nlm.nih.gov/sra) under accession number PRJNA374530.

**Table 1:** Summary of the RNA-Seq data of both *Anisakis simplex* (*s.s.*) and *Anisakis pegreffii* whole third-stage larvae (WL) and oesophageal-pharyngeal region (PX). For *A. pegreffii*, assembly and results of bioinformatic analyses and annotation are reported. For both *A. simplex* and *A. pegreffii*, upregulated differentially expressed genes (DEGs) are also reported

	WL	PX
*Anisakis simplex* (*s.s.*)		
Number of reads	32,859,191	37,606,372
Number of reads mapping against genome	29,557,642	34,029,700
Number of uniquely mapping	28,085,077	32,380,240
Number of Mbp	1369	1567
DEGs	109	226
*Anisakis pegreffii*		
Number of reads	23,397,005	36,382,470
Number of Mbp	975	1515
*De-novo* assembly		
Number of transcripts	21,195^a^	
Mean length	468	
N50	2063	
Annotation		
Number of ORFs	10,424	
Number of transcripts matching available sequences in Nr	7920	
Returning SwissProt	8803	
Gene Ontology	8560	
Kyoto Encyclopoedia of Genes and Genomes	7369	
DEGs	163	339

^a^Transcripts obtained from pooled reads

Given the availability of a draft genome sequence for *A. simplex* [31] (Wormbase BioProject PRJEB496; http://parasite.wormbase.org/Anisakis_simplex_prjeb496/Info/Index/), raw reads generated for AS were mapped to the existing genome assembly sequences (Wormbase BioProject PRJEB496); approximately 90% of reads for AS-WL and AS-PX could be mapped to the available genome sequences, with 86% of these mapping only once. The 61,865,213 pooled reads obtained from both WL and PX of AS, mapped to a total of 19,403 genes (out of 20,971) in the AS genome. Conversely, due to the unavailability of a draft genome sequence for AP, raw reads generated from WL and PX samples for this species were assembled *de novo* (see Methods). The pooled 53,839,897 reads for AP-WL and AP-PX yielded, following quality-filtering, 21,195 contigs (Table 1). Shared transcripts (putative orthologs) between AS and AP were identified by pairwise BLASTP reciprocal best hit analysis (E-value cut-off: 1e-06; minimum hit coverage 30%), performed on the longest ORF identified for each transcript, resulting in a list of 4635 putative orthologs (Additional file 1: Table S1).

### Transcript annotation and differential gene expression analysis

Annotation information linked to AS transcript-encoding genes were derived from data available from the WormBase repository database at http://parasite.wormbase.org/Anisakis_simplex_prjeb496/. For AP, a total number of 10,424 predicted peptides could be inferred from the 21,195 assembled transcripts (Table 1). Of these transcripts, ~35% could be annotated via BLAST searches against the nr, SwissProt, KEGG and GO databases (Table 1). Detailed annotation information for individual AP transcripts identified in this study is available in Additional file 2: Table S2.

For each AS and AP, complete lists of differentially expressed genes (DEGs) in WL and PX containing annotations informations are available in Additional file 3: Table S3 and Additional file 4: Table S4.

The search of signal peptides in both pharyngeal and whole larva enriched catalogues was carried out with the aim to better define the secretory nature of predicted proteins. In both AS and AP, the pharynx and the whole larva subsets (Table 2) were therefore sorted to retrieve only peptides starting with Met and the probability to carry signal peptides was evaluated using the SignalP Server (Table 2). Putative secreted proteins were detected in transcripts upregulated in the PX of each AS and AP (20 and 36% of the total number of upregulated transcripts, respectively) pointing out a secretory aptitude of pharyngeal repertoires. Totals of 16 and 22% of transcripts upregulated in WL of each AS and AP were predicted to encode a signal peptide.

**Table 2:** Signal peptides predictions. Number of transcripts encoding signal peptides predicted in *Anisakis simplex* (*s.s*.) and *Anisakis pegreffii* differentially expressed transcripts (WL, whole larva; PX, pharynx). The percentages of putatively secreted peptides over the total number of transcripts upregulated in the WL and PX of each species are also indicated

	*Anisakis pegreffii*		*Anisakis simplex* (*s.s.*)	
	PX-UP (140)	WL-UP (113)	PX-UP (226)	WL-UP (109)
Peptides (Met)	76	55	174	75
SP	27	12	35	12
% SP	36	22	20	16

Pfam enrichment analyses were also performed in both AS and AP by comparing the pharyngeal upregulated subset (FDR < 0.05) with the whole transcript sequence datasets (Table 3).

**Table 3:** Protein families (according to information in the Pfam database) identified amongst the upregulated transcripts in the pharynx (PX) of *Anisakis simplex* (*s.s.*) and *Anisakis pegreffii.* Pfam code and description, relative abundance of transcripts compared to the whole transcriptome (expressed as ratio between relative frequencies) and medial logFC are reported

PFAM	PFAM description	PX enrichment	Median logFC
*Anisakis simplex* (*s.s.*)			
PF00188	Cysterin-rich secretory protein	9.71	2.17
PF01400	Astacin peptidase M12A	16.19	2.15
PF01431	Peptidase M13	11.88	2.48
PF01433	Peptidase M1	2.48	2.68
PF11838	Peptidase active site	17.81	2.68
PF00450^a^	Peptidase S10	3.23	2.42
PF01549	ShK toxin	13.36	2.23
PF00014^a^	Kunitz/Bovine pancreatic trypsin inhibitor domain	4.97	2.65
PF01683	EB module	18.59	2.55
PF00031	Cystatin	13.36	2.06
*Anisakis pegreffii*			
PF00188	Cysterin-rich secretory protein	9.29	3.41
PF01400	Astacin peptidase M12A	12.62	3.28
PF01431	Peptidase M13	13.59	3.02
PF01433	Peptidase M1	5.52	3.66
PF01549	ShK toxin	8.83	2.75
PF01683	EB module	58.88	2.98
PF00063	Myosin head motor domain	11.39	2.71
PF00031	Cystatin domain (cysteine protease inhibitor)	8.83	2.81
PF06394	Pepsine inhibitor like	8.03	3.14
PF14580	Leucine-rich	88.31	4.93

^a^Pfam associated to transcripts detected in AS-PX only

**Fig. 2:**
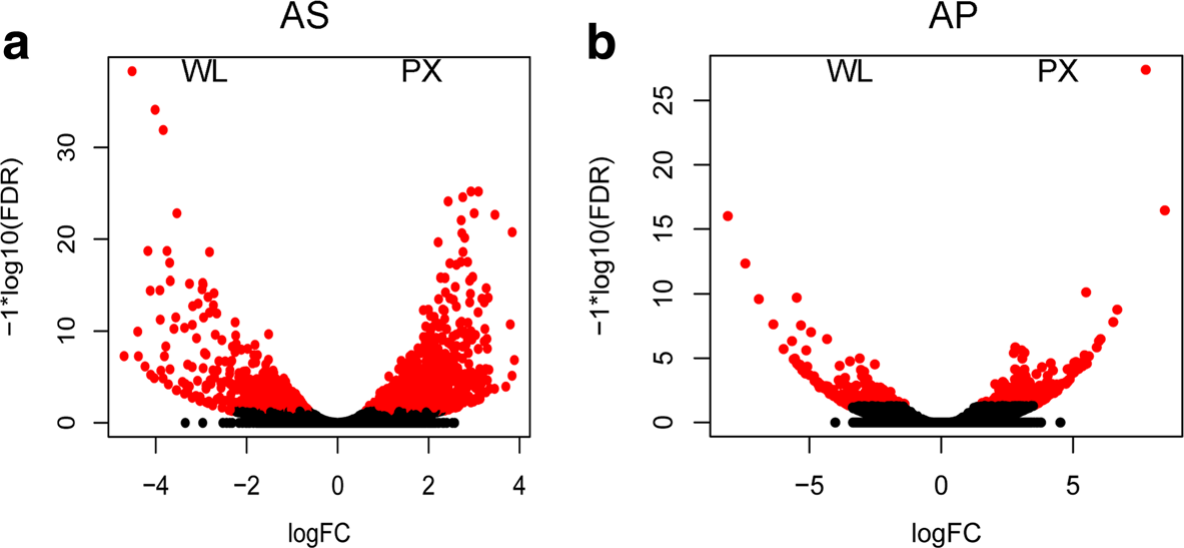
Tissue-specific gene expression. Volcano plots displaying the relative expression levels of transcripts upregulated in the pharynx (PX) compared with the whole larva (WL) in **a** *Anisakis simplex* (*s.s.*) (AS) and **b** *Anisakis pegreffii* (AP). The x-axis represents the log2 of the expression ratio for each transcript (tissue specific logCPM: whole body counts per million reads, logCPM, CPM); the y-axis represents the log10 of the *P*-value corrected for the false discovery rate. Red dots represent differentially expressed transcripts with logFDR < 0.05 (at least 2-fold difference in logCPM). Positive logFC values indicate transcripts enhanced in the pharynx subset, while negative values indicate transcripts upregulated in the whole larva

For AS, a total number of 335 differentially expressed genes were identified (Fig. 2a), of which 109 were upregulated in WL and 226 in PX (Additional file 2: Table S2, Additional file 4: Table S4). Seventy-one and 107 of these genes, respectively, were annotated with Pfam information (Additional file 2: Table S2, Additional file 4: Table S4). The remaining genes could not be annotated based on the information available in current public databases. Transcripts upregulated in WL encoded mostly enzymes with proteolytic activity, e.g. peptidases M1 and S10 (Additional file 2: Table S2). In the PX subset, 34.5% contigs encoded for proteins with putative roles in worm metabolism and physiology, including sugar transport, nervous system and motility. This reflects the biological function of this anatomical region, which includes, for instance, the nervous ring and the digestive oesophagous (see Fig. 1). A further 16% (36/226) of transcripts upregulated in the PX of this species encoded for (i) peptidases; (ii) members of the CAP superfamily, including proteins similar to known allergens from the venom of wasps, honeybees (such as histidine phosphatase superfamily members and cysteine-rich secretory proteins) and snakes (such as those carrying a pancreatic trypsin inhibitor Kunitz domain); and (iii) molecules with putative immune-modulatory function (e.g. leucine-rich repeat; immunoglobulin subtype, immunoglobulin-like domain, immunity-related GTPases-like) (Table 3). In particular, putative peptidases included (i) aspartic peptidases M1 (*n* = 1), (ii) astacin peptidase M12A (*n* = 5), (iii) peptidase M13 (*n* = 2), (iv) serine carboxypeptidase S10 (*n* = 1), as well as hemopexin-like metallopeptidases, carboxylesterases and ShKT *Stichodactyla helianthus* toxin (*n* = 3). Other predicted proteins upregulated in the PX subset with similarity to known allergens included molecules encoding for cysteine-rich domains, such as Kunitz, lustrins, trypsin inhibitors and ‘*Clostridium* epsilon toxin’; the latter was only detected only in the PX subset of AS (Table 3 and Additional file 1: Table S1).

A total of 502 DEGs were identified in AP, of which 163 were upregulated in WL and 339 in PX (Fig. 2b and Additional file 3: Table S3, Additional file 4: Table S4). Of these, 80 and 88 could be annotated with Pfam terms, while 83 and 100, respectively, matched amino acid sequences available in the NCBI nr database (Additional file 3: Table S3, Additional file 4: Table S4).

Amongst transcripts upregulated in the PX subset, 43 (48%) encoded for proteins with putative structural and motility functions (i.e. myosins and tropomyosins) or playing roles in worm physiology and metabolism. Similarly to AS, transcripts encoding for (i) peptidases, (ii) members of the CAP superfamily, and (iii) “toxins” were enriched in AP-PX subset. In particular, peptidases M1 (*n* = 1), M12A (*n* = 6) and M13 (*n* = 1), members of the CAP superfamily (*n* = 8) and peptides containing ‘ShK toxin’ domains (*n* = 2) were identified (Additional file 2: Table S2).

GO terms enrichment analysis was performed to compare groups of transcripts upregulated in the WL and PX of each AS and AP (cf. Additional file 5: Figure S1 and Additional file 6: Figure S2, respectively).

**Fig. 3:**
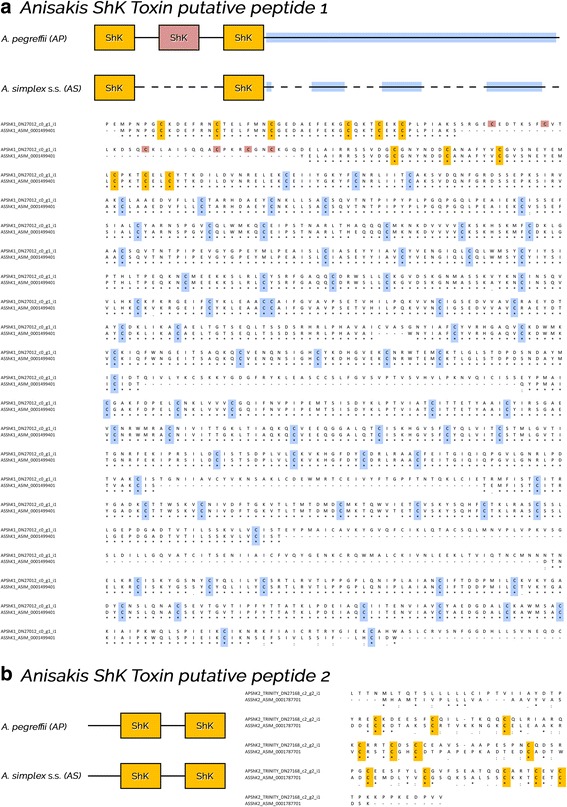
Organization and alignment of two ShK toxin putative peptides (**a**, **b**) in *Anisakis pegreffii* (AP) and *A. simplex* (*s.s.*) (AS). ShK-cysteines are highlighted in yellow and light red; cysteine-rich domain in light blu. Identical residues, conservative and semi-conservative substitutions are indicated with an asterisk, colon and semicolon, respectively

Putative orthologous transcripts encoding for proteins upregulated in the PX of both AS and AP, of interest based on their putative biological function in the tissues analysed, were characterized in further detail. Two transcripts in AP and one in AS encoded for putative ShK toxin peptides, with different domain organization as revealed by amino acid sequences alignments (Fig. 3). The first ShK peptide identified in AP (AP1) contained three adjacent ShK domains followed by a cysteine-rich portion at the N-terminus, in contrast to AS predicted ortholog that contain only two ShK domains. The second putative ShK peptide contained two adjacent domains in both AS and AP (Fig. 3). Interestingly, the C-terminus of *ShKgene1* partially matched known allergens of *A. simplex* (*s.l.*), as revealed by BLAST analysis (Anis 7 GenBank accession number ABL77410, query coverage 93% and identity 25%; Anis 12 GenBank accession number AGC60031, query coverage 46% and identity 33%; Anis 14 GenBank accession number BAT62430, query coverage 71% and identity 34%). A transcript encoding for a similar ShK C-term could not be identified in AS using the Artemis tool, likely as a result of the current fragmentation of available genomic scaffolds.

**Fig. 4:**
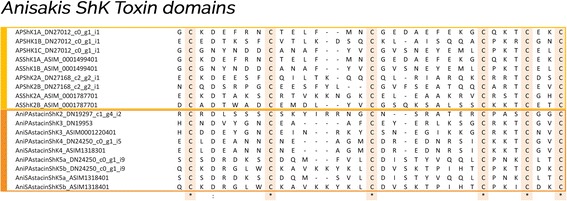
Alignment of putative ShK toxin domains in *Anisakis pegreffii* (AP) and *A. simplex* (*s.s.*) (AS). ShK-cysteines are highlighted in light orange. Yellow box indicates ShK domains of ShK containing transcripts; orange box indicates astacin associated ShK domains. Identical residues, conservative and semi-conservative substitutions are indicated with an asterisk, colon and semicolon, respectively

**Fig. 5:**
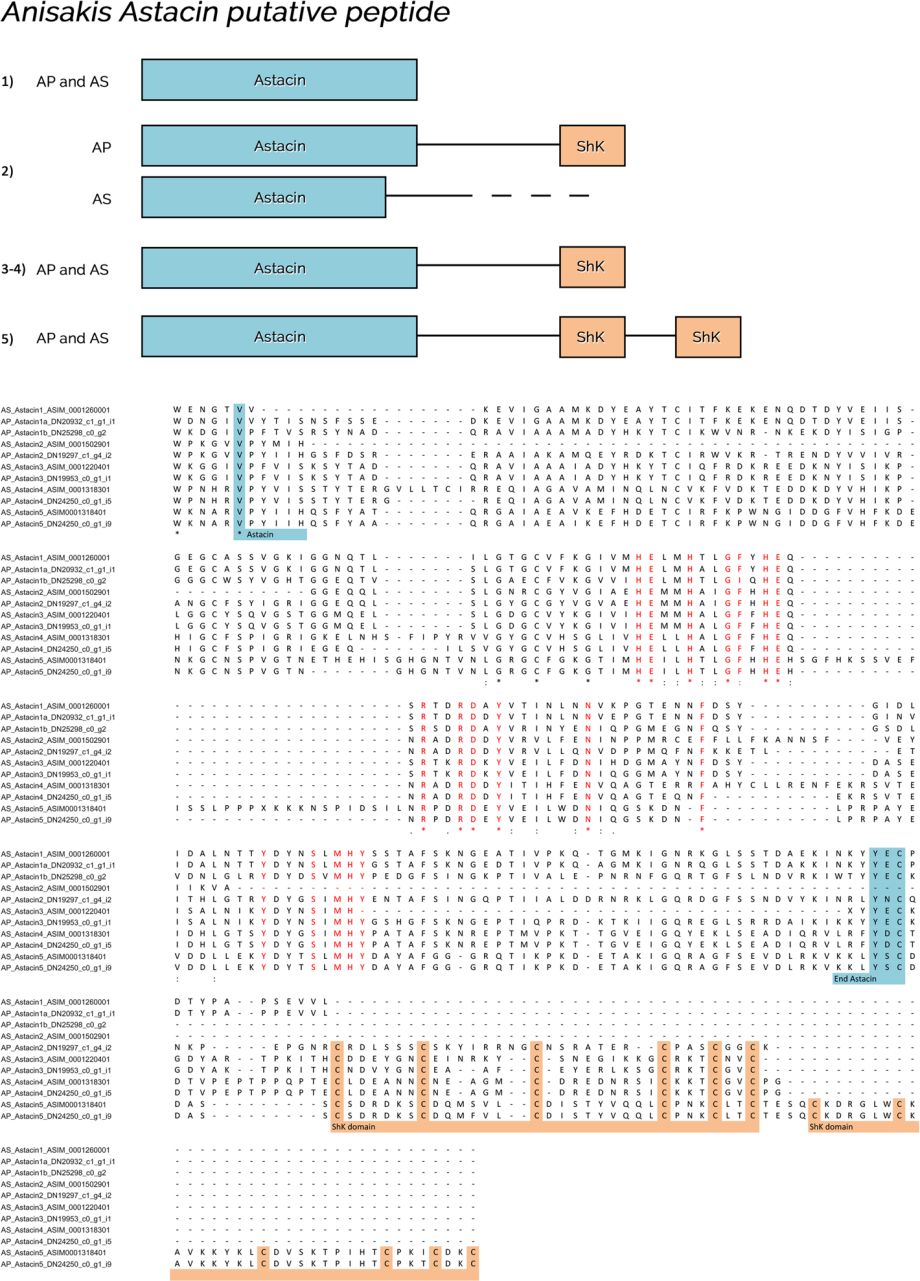
Alignment and organization of five putative astacin metalloendopeptidases of *Anisakis pegreffii* (AP) and *A. simplex* (*s.s.*) (AS). The astacin module is indicated in light blue boxes, while orange boxes indicate astacin associated ShK domains

ShK toxin domains were also found associated to astacin peptides (Fig. 4). In particular, five putative orthologous peptides encoding for astacin metalloendopeptidase were identified in AS and AP, with a different number of ShK modules (one or two, Fig. 5). Such modular structures were observed in other organisms: while 38% of sequences with only astacin domain retrieved in the Pfam database belonged to parasitic nematodes, around 80 and 83% of sequences with astacin domain associated to one or two ShK modules, respectively, were represented by parasitic nematodes.

Among transcripts encoding for cysteine-rich proteins, orthologous members of the CRISP-SCP family (CAP-superfamily) were detected in both AS and AP (Additional file 7: Figure S3). In particular, one transcript significantly upregulated in the PX of both species encoded for typical conserved residues of SCP-like proteins. Conversely, no differential expression was detected for the remaining transcripts encoding for the other three CRISP orthologues (alignments of peptides are available in Additional file 7: Figure S3).

Transcripts encoding putative proteins containing an EB module, often associated with Kunitz domains, were upregulated in the PX of both AS and AP: two orthologues were identified, with a further paralogue in AS (Additional file 8: Figure S4). In particular, five EB modules were identified in AP1 (*Anisakis* EB module 1), four in AS_EB1A and three in AS_EB1B (Additional file 8: Figure S4a) whereas, for *Anisakis* EB module 2, four adjacent EB modules were observed in both AP and AS (Additional file 8: Figure S4b).

Finally, transcripts encoding putative peptides carrying Kunitz domains were enriched in AS PX only. Two putative peptides, both containing conserved a cysteine-rich region known as lustrin, showed homology to “major allergen” Anis1 found in *Anisakis*, *Ascaris* and *Toxocara* (Additional file 9: Figure S5). One of the two peptides encompasses two Kunitz domains followed by two lustrin domains (Additional file 9: Figure S5a), while the second contained seven repeates of lustrin-Kunitz domains (Additional file 9: Figure S5b).

## Discussion

Parasitic nematodes infect more than one billion people globally, causing substantial suffering and massive economic losses [32]. Anisakidosis is an emerging fish-borne zoonosis of major public health concern. Nevertheless, the impact of the disease is largely underestimated and our current understanding of the mechanisms of infection and clinical disease in humans is still scant.

While knowledge of the complex mechanisms underlying host-pathogen interplay in several parasitic nematodes is expanding [33–35], studies of the interactions between *Anisakis* and its accidental hosts are in their infancy [18, 19]. Previous evidence suggested that secretions from the pharyngeal subventral and esophageal dorsal glands of the parasites may play roles in the intraluminal and extracorporeal digestion of tissues of natural (intermediate or paratenic) hosts [21] and in the invasion of the gastrointestinal mucosa of human accidental hosts [21].

In an effort to investigate the nature and the potential role(s) of molecules expressed by the pharyngeal tissues of *Anisakis* spp. in pathways linked to pathogenicity, we analyzed the whole larval and pharyngeal transcriptomes of the two pathogenic sibling species AS and AP*.* The analysis of transcriptomes provided a number of transcripts similar to those inferred from previous studies of *Anisakis* spp. and of other non-model species of parasitic nematodes [19, 35, 36]. In addition, several shared transcripts were identified in these two species, albeit the total number of orthologous genes may be underestimated due to the unavailability of a draft genome sequence for *A. pegreffii.* The number of full-length peptides predicted from assembled transcript sequences for AP was significantly lower than the corresponding number for AS, thus indicating that a significant number of AP transcripts could not be assembled with confidence.

Nevertheless, comparative analyses of levels of gene transcription in the WL and PX of these two species led to the identification of groups of molecules with putative roles in mechanisms of parasite pathogenicity. Amongst these molecules, the cysteine-rich secretory proteins (CRISPs), belonging to the CAP superfamily (cysteine-rich secretory proteins, antigen 5, and pathogenesis-related 1 proteins), were signifincatly upregulated in the PX of both AS and AP. Members of this family are secreted proteins with extracellular endocrine or paracrine functions, identified in several eukaryotes, including arthropods, flatworms and roundworms (reviewed by Cantacessi et al. [37]). In nematodes, members of this family are also known as *Ancylostoma*-secreted proteins or activation-associated secreted proteins (ASPs) (reviewed by Cantacessi et al. [37]). Increased transcription of ASPs is observed during the transition of the ensheathed, free living L3 of the dog hookworm, *Ancylostoma caninum*, to its exsheathed parasitic form [38]. While the exact function(s) of nematode ASPs are yet to be uncovered, this observation suggests that these molecules may be involved in biological pathways associated with penetration of host tissues and/or interactions with the host immune system (cf. [35, 36]).

A group of proteins with roles in host tissue penetration and digestion encoded by transcripts were differentially expressed in the PX of both AS and AP is represented by the peptidases. In particular, transcripts encoding for metalloproteinases (i.e. aminopeptidases, astacins and neprilysins) were particularly abundant in AS. Aminopeptidases catalyze the removal of single amino acids from the amino N-terminus of small peptides, thus playing a role in their final digestion [39]. In addition, members of this family hydrolyse the epoxide leukotriene-A4 to form an inflammatory mediator [40]. The *Anisakis* orthologue might be involved in eliciting a host immune and/or inflammatory response. Astacins are implicated in the activation of growth factors, degradation of polypeptides, and processing of extracellular proteins, also sharing common features with serralysins, matrix metalloendopeptidases, and snake venom proteases [40]. Astacins might also play a role as anticoagulants; indeed, the hematophagus mollusk *Colubraria* secretes Astacin/ShKT-containing proteins that may inhibit ion channels, interfere with hemostasis and facilitate the spreading of the toxins in the body of the prey by degrading extracellular matrix molecules and inactivating several endogenous vasoactive peptides [41]. In the free-living nematode *Caenorhabditis elegans*, an astacin enzyme is expressed in the hypodermal tissue and is required for normal collagen secretion [42]. Astacins are also found in the parasitic filarioid *Brugia malayi* and in the trichostrongylid *Haemonchus contortus* [43] where they play a role in collagen processing and cuticle formation. Proteases belonging to the M13 family include, amongst other proteins, the neprilysins, molecules found in a wide range of vertebrates (including mammals), and invertebrates (e.g. *Drosophila* and *C. elegans* [44, 45]). In mammals, these proteins are involved in cardiovascular development, blood-pressure regulation, nervous control of respiration, and regulation of neuropeptides in the central nervous system, while their biological functions in nematodes are yet unclear. However, in experimentally infected mice, M13 peptidases secreted by *Trichinella spiralis* have been demonstrated to participate in the early onset of intestinal inflammation [46]. Similar mechanisms may occur over the penetration of intestinal tissues by *Anisakis* larvae. Amongst other peptidase-encoding transcripts upregulated in the PX of both AS and AP and with putative roles in host tissue digestion, serine carboxypeptidases have been previously detected in *Angiostrongylus cantonensis* [47] and *Strongyloides ratti* [48] where, similarly to aminopeptidases, they also serve as digestive enzymes.

Molecules encoding for potential anaesthetics were also detected amongst transcripts differentially expressed in the PX of both AS and AP, including predicted peptides containing a ShK toxin domain. In venomous organisms, the ShKT is a signature of potent ion channel blockers [49], whereas the presence of this protein motif in oxidoreductases and prolyl hydroxylases of plants, and in astacin-like metalloproteases and trypsin-like serines proteases of helminths confers these enzymes potential roles in potassium channel-modulatory activities. However, in the zoonotic dog roundworm *Toxocara canis*, secreted mucins implicated in immune evasion mechanisms are characterized by the presence of evolutionarily distinct modules, i.e. the mucin and ShKT domains [50], which raises questions on the possible roles on *Anisakis* ShKT domain-encoding molecules in interactions between the parasite and the host immune system. Moreover, the association of astacin and ShK toxin domain appears to be particularly common in parasitic nematodes.

Besides transcripts of interest detected in both AS and AP, a relatively small number of molecules were exclusively upregulated in the PX of AS. These molecules included peptides encoding Kunitz domains, with trypsin inhibitor activity, and aspartic proteases. Proteins with Kunitz domains are degrading enzymes that act as protease inhibitors, with a functionally conserved role in cuticle formation in a diverse range of nematodes [43]. In blood-sucking organisms such as ticks [51, 52] and molluscs [41], proteins containing Kunitz domains are possibly involved in the inhibition of thrombin and coagulation factors. Functions in mechanisms aimed to prevent attack by host proteolytic enzymes and coagulation factors in the host blood stream are suggested for the zoonotic tapeworm *Echinococcus granulosus* [53], and for the blood flukes *Schistosoma japonicum* and *S. mansoni* [54, 55]. Excretory/secretory products from *A. simplex* (*s.l.*) larvae contain molecules with anticoagulant activities that may play a role in the pathogenesis of anisakiasis in accidental hosts [56]. In *Anisakis*, proteins with Kunitz domains with trypsin-like proteinase properties have been previously identified in studies examining the proteolytic enzymatic activity of this parasite [20, 57, 58]; their optimal activity was recorded at pH 7.5 (approximately the pH of the terminal ileum) and a host body temperature of 36–37 °C, thus supporting the hypothesis that these enzymes are activated during invasion of the intestine in the homeothermic hosts. In addition, one of the major known *Anisakis* allergens, i.e. Anis1, contains a Kunitz-domain [59]. A transcript upregulated in AS PX showed high homology to this antigen (Additional file 9: Figure S5a, b). Indeed, it is possible to hypothesize that novel molecules other than the already known peptides with antigenic and pathogenic properties may elicit the inflammatory and immune response observed in humans. Aspartic proteases (pepsin-like) were also included amongst the upregulated AS molecules. The best known source of aspartic proteases is the stomach of mammals [60], and pepsin was identified as a positive regulator of cathepsin-B involved in the penetration of rat gut by *Angiostrongylus cantonensis* [60, 61]. Results from our study suggest that transcripts encoding selected Kunitz-domain containing proteins and aspartic proteases were exclusively expressed by AS. However, given that the set of AP transcripts were assembled *de novo* in the absence of a genome reference sequence, and the significantly lower number of predicted peptides, their presence in the genome and transcriptome of AP cannot be fully excluded.

## Conclusions

By undertaking the first large-scale analysis of molecules expressed in the PX of *Anisakis*, i.e. the organ putatively involved in host recognition/adhesion/invasion, we herein provide the community with a ready-to-use molecular groundwork for in-depth studies of biological pathways specifically involved in host-parasite interplay. The identification of species- and/or tissue-specific molecules in two *Anisakis* species provides new information on the expression of genes potentially involved in host tissue invasion. The results obtained may assist in the characterization of properties or functions of these molecules, and their exploration as potential novel targets for treatments and control strategies [62]. Further investigations using experimental in vitro controlled conditions as well as differential gene expression between species and in other developmental life cycle stages, are needed to reliably asses the roles of these moleculesin the pathogenesis of anisakiasis.

## Additional files

Additional file 1: Table S1. Putative orthologs of *Anisakis simplex* (*s.s.*) (AS) identified in the transcriptome of *Anisakis pegreffii* (AP) and *vice versa*. (XLS 3657 kb)

Additional file 2: Table S2. Annotation information of *Anisakis simplex* (*s.s*.) (AS) differentially expressed genes (DEGs) in the whole larva (WL) and in the pharynx (PX). Corresponding peptide sequences are also included. (XLSX 9154 kb)

Additional file 3: Table S3. *Anisakis pegreffii* transcript sequences and, where available, corresponding peptides and annotation information, and differentially expressed genes (DEGs) upregulated in the whole larva (WL) and in the pharynx (PX). (XLSX 170 kb)

Additional file 4: Table S4. Data including TPM and FDR of *Anisakis simplex* (*s.s.*) (AS) and *Anisakis pegreffii* (AP) differentially expressed genes (DEGs) in the whole larva (WL) and in the pharynx (PX). (XLSX 197 kb)

Additional file 5: Figure S1. Gene Ontology term distribution of differentially expressed genes (DEGs) in *Anisakis simplex* (*s.s.*). (JPEG 579 kb)

Additional file 6: Figure S2. Gene Ontology term distribution of of differentially expressed genes (DEGs) in *Anisakis pegreffii*. (JPEG 982 kb)

Additional file 7: Figure S3. Alignment of four orthologous putative peptides belonging to CRISPs (CAP superfamily) in *Anisakis simplex* (*s.s.*) (AS) and *A. pegreffii* (AP). (TIFF 674 kb)

Additional file 8: Figure S4. Alignment of two EB-module containing orthologues in *Anisakis simplex* (*s.s.*) (AS) and *A. pegreffii* (AP), indicated as module 1 and 2. Conserved cysteines of EB domain are highlighted in red and the whole domain is highlighted in violet. (TIFF 2315 kb)

Additional file 9: Figure S5. Alignement of *Anisakis simplex* (*s.s.*) (AS) putative peptide containing Kunitz domain (**a**: ASIM_0001030201 and **b**: ASIM_0001813801) with sequences of major allergens AniS1 of *A. simplex* (AGC60037), *A. pegreffii* (AGC60032), *Toxocara canis* (KHN76536), *Ascaris suum* (ERG81619), *Trichuris trichiura* (CDW53412), *Necator americanus* (XP_013296851) *Brugia malayi* (XP_001901728), *T. canis* (KHN72724), *A. suum* (ERG79727), *Strongyloides ratti* (CEF68915) and *Caenorhabditis elegans* (NP_001256870). (JPEG 201 kb)

## Abbreviations

AP: *Anisakis pegreffii*
AS: *Anisakis simplex* (*s.s.*)
ASPs: *Ancylostoma*-secreted proteins or activation-associated secreted proteins
CAP: Superfamily of proteins (cysteine-rich secretory proteins, antigen 5, and pathogenesis-related 1 proteins)
CPM: Count per million reads
CRISPs: Cysteine-rich secretory proteins
DEGs: Differentially expressed genes
FDR: False discovery rate
L3s: *Anisakis* third-stage larvae
Mbp: Megabase pairs
Pfam: Protein family
PX: Pharyngeal region
TMM: Trimmed mean of M-values normalization method
TPM: Transcripts per million
WL: Whole larva
