# Supplementary figures and images for: Tissue-specific transcriptomes of Anisakis simplex (sensu stricto) and Anisakis pegreffii reveal potential molecular mechanisms involved in pathogenicity
Source: Parasit Vectors. 2018 Jan 10;11:31. doi: 10.1186/s13071-017-2585-7 (PMC5763927; doi:10.1186/s13071-017-2585-7)

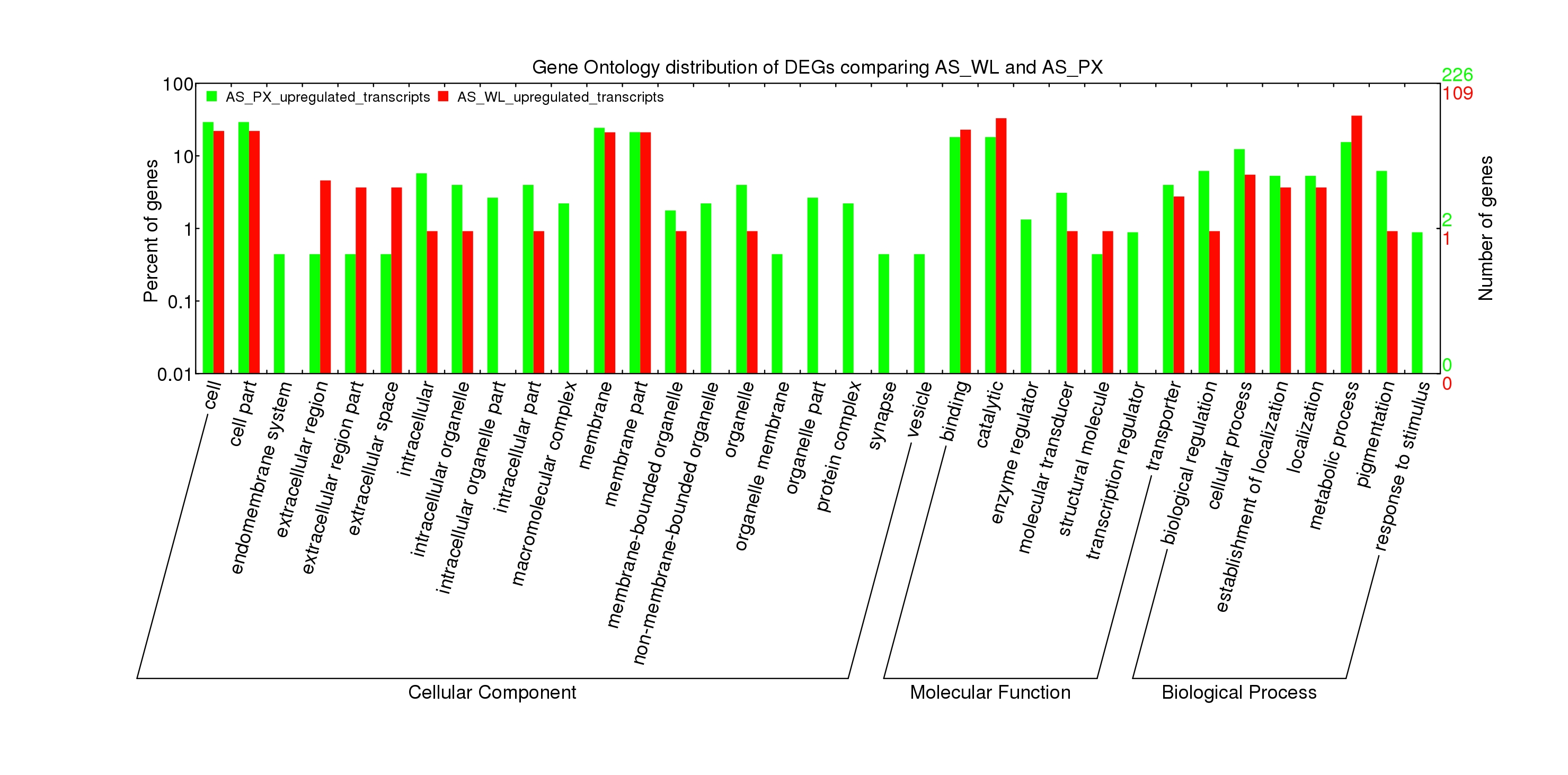

Supplement: Supplementary file 5 — Gene Ontology term distribution of differentially expressed genes (DEGs) in Anisakis simplex (s.s.). (JPEG 579 kb) [file 13071_2017_2585_MOESM5_ESM.jpg]

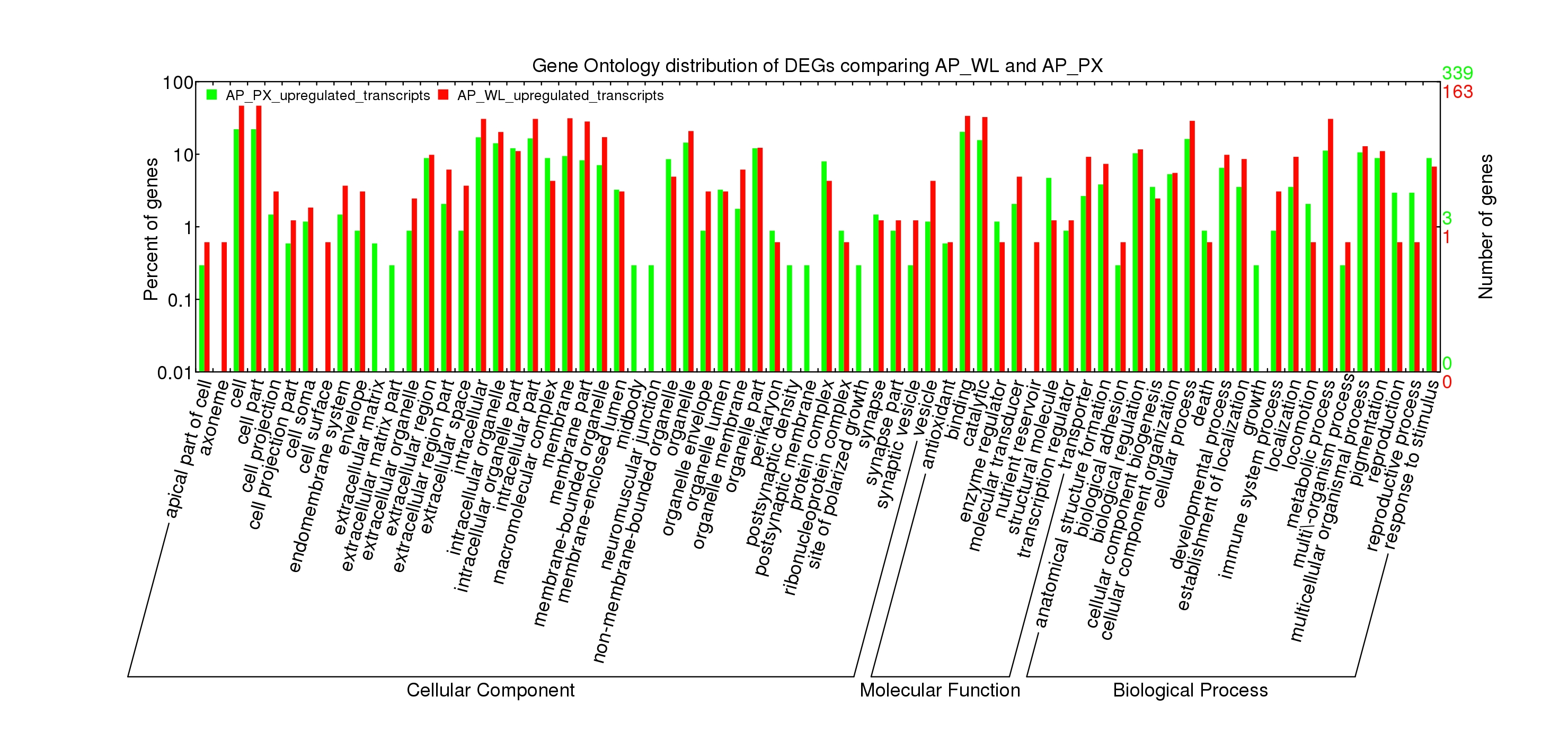

Supplement: Supplementary file 6 — Gene Ontology term distribution of of differentially expressed genes (DEGs) in Anisakis pegreffii. (JPEG 982 kb) [file 13071_2017_2585_MOESM6_ESM.jpg]

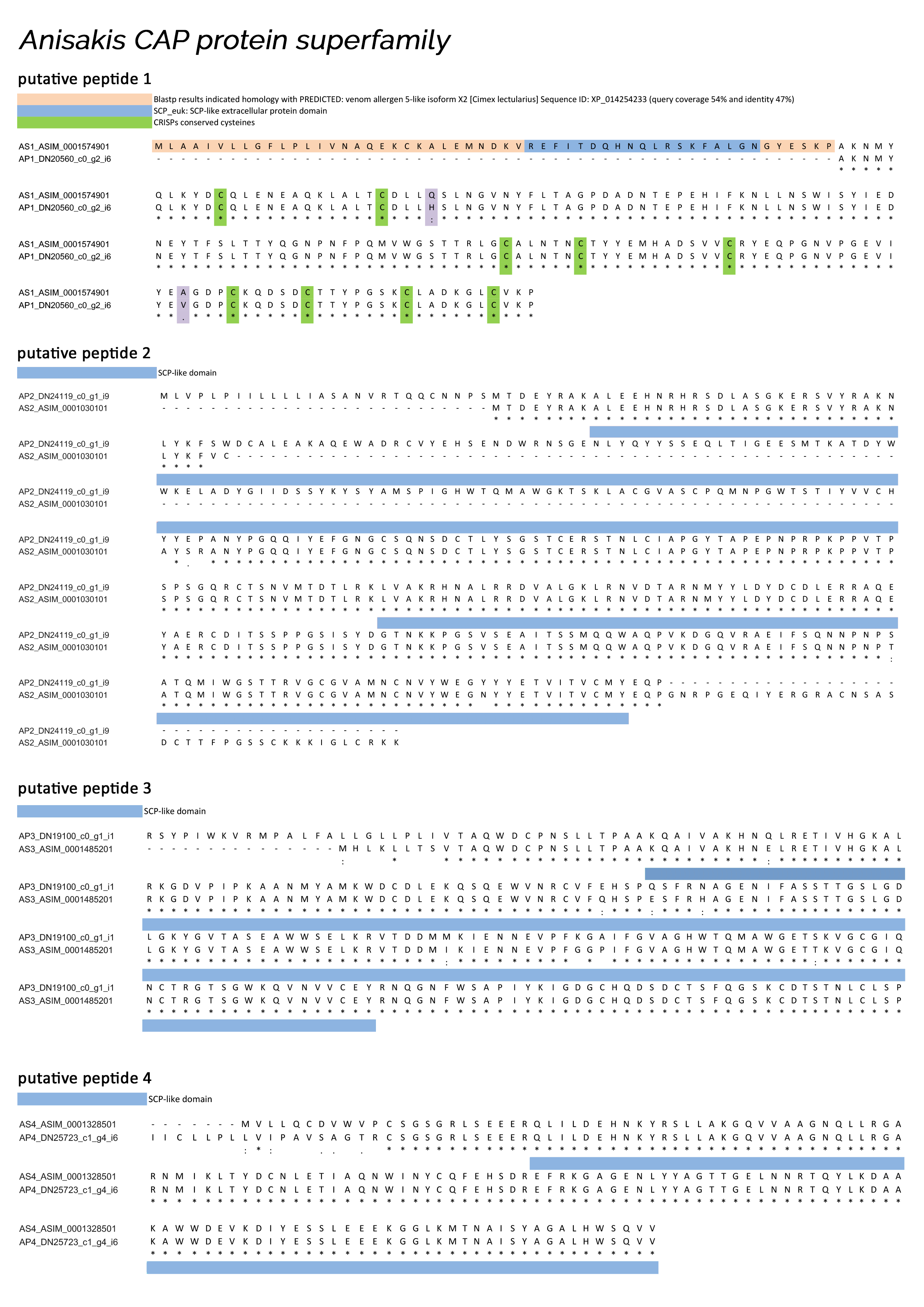

Supplement: Supplementary file 7 — Alignment of four orthologous putative peptides belonging to CRISPs (CAP superfamily) in Anisakis simplex (s.s.) (AS) and A. pegreffii (AP). (TIFF 674 kb) [file 13071_2017_2585_MOESM7_ESM.tif]

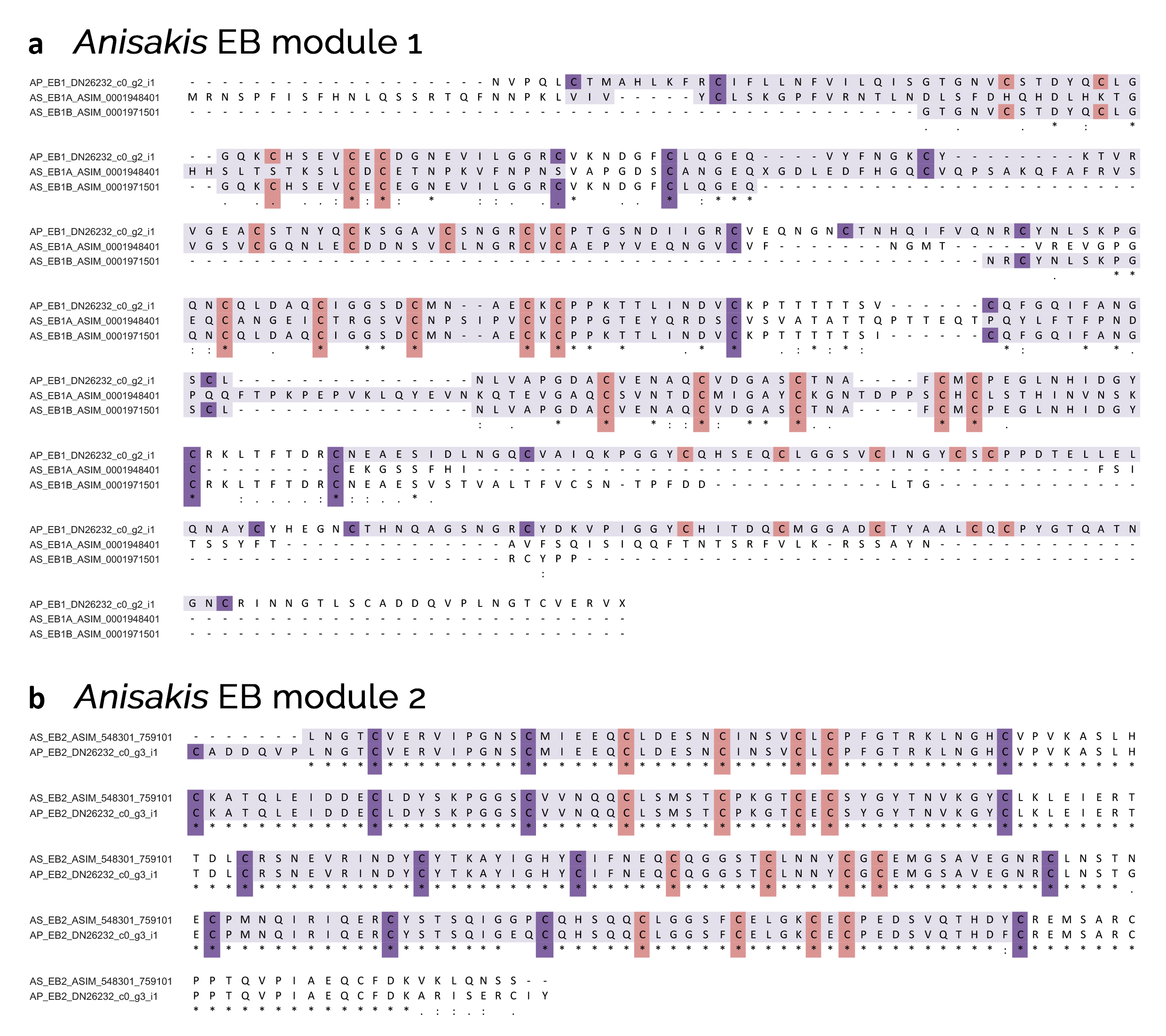

Supplement: Supplementary file 8 — Alignment of two EB-module containing orthologues in Anisakis simplex (s.s.) (AS) and A. pegreffii (AP), indicated as module 1 and 2. Conserved cysteines of EB domain are highlighted in red and the whole domain is highlighted in violet. (TIFF 2315 kb) [file 13071_2017_2585_MOESM8_ESM.tif]

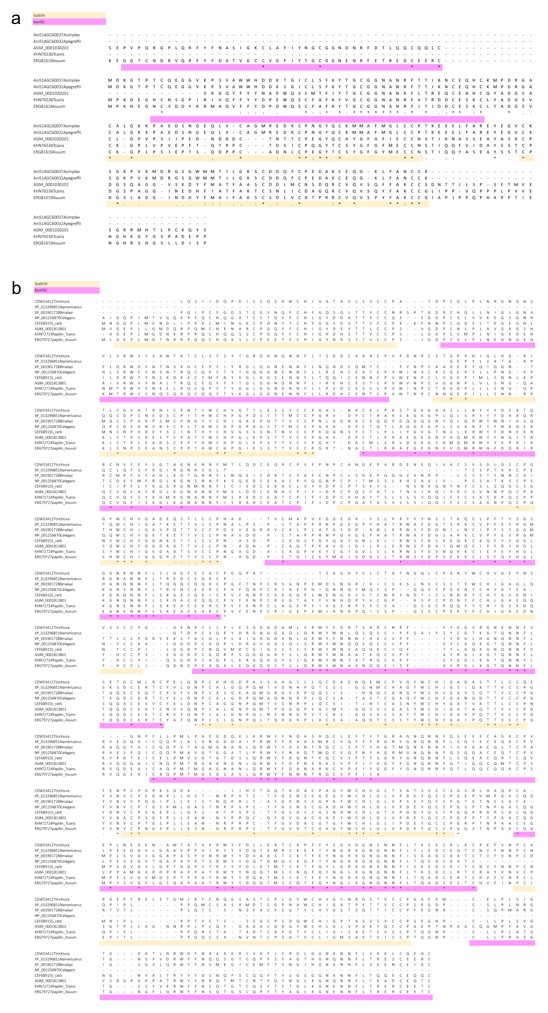

Supplement: Supplementary file 9 — Alignement of Anisakis simplex (s.s.) (AS) putative peptide containing Kunitz domain (a: ASIM_0001030201 and b: ASIM_0001813801) with sequences of major allergens AniS1 of A. simplex (AGC60037), A. pegreffii (AGC60032), Toxocara canis (KHN76536), Ascaris suum (ERG81619), Trichuris trichiura (CDW53412), Necator americanus (XP_013296851) Brugia malayi (XP_001901728), T. canis (KHN72724), A. suum (ERG79727), Strongyloides ratti (CEF68915) and Caenorhabditis elegans (NP_001256870). (JPEG 201 kb) [file 13071_2017_2585_MOESM9_ESM.jpg]
